# The politics of dispossession and compensation in the eastern Indian coal belt

**DOI:** 10.1177/0308275X221074831

**Published:** 2022-02-07

**Authors:** Itay Noy

**Affiliations:** Centre for Energy Ethics and Department of Social Anthropology, University of St Andrews, London, UK

**Keywords:** Adivasi, class, compensation, dispossession, mining, politics

## Abstract

Ethnographic studies in sites of land dispossession for large capital projects have revealed the diversity of local political responses to this process, from fierce resistance to compliance. The theoretical challenge, in this context, is to trace the particular factors that affect this politics, and the conditions under which different reactions to dispossession unfold. Drawing on fieldwork in an Adivasi (tribal) village adjacent to an opencast coal mine in Jharkhand, India, this article seeks to contribute to this inquiry. It illustrates how, in a predominantly precarious labour environment, the possibility of formal employment as compensation for expropriated land, and the ways in which such employment enables class mobility, can play a salient role in shaping local political dynamics around dispossession. The analysis shows how, in the community studied, compensatory jobs for dispossession gave rise to new class differentiation and shifts in political relations that have acted to curb potentialities of resistance – precisely in a context in which opposition to dispossession could have otherwise been considered likelier to emerge.

Shiv^1^ and his wife Savitha left their mud house before dawn and set out on foot to the colliery near the village, as they did almost every morning. Taking advantage of the fact that the security guards had yet to arrive, Savitha entered the mine’s depot yard and started gathering lumps of coal, using the metal bowl she had brought with her. Shiv stayed behind, on a patch of land at the edge of the mine. Using large jute bags, he started packing the coal Savitha had collected on previous days, which was piled in a heap in front of him. Later, Shiv would tie the bags onto his bicycle and set off to the main road to sell the coal to restaurants and informal petty coal brokers, which is how he and Savitha made their living. Meanwhile, on the other side of the village, Ramesh’s day was off to a very different kind of start. He slipped into his fake leather shoes, left his concrete built house, and got on his motorbike to ride to the colliery, where he has a permanent job in the machinery workshop. The dirt road to the workshop took him around the depot, where Shiv, Savitha and other villagers were still toiling away, their hands and feet covered in a thick layer of coal dust.

This typical morning in the Adivasi, or tribal, village of Karampot, in the coal-bearing tracts of Jharkhand, eastern India, encapsulates some of the changes brought about by the arrival of mining operations in its vicinity. Inhabited predominantly by Adivasis from the Santal tribe, the largest in the state, Karampot is located next to Pandu Project, an opencast colliery run by the state-owned Central Coalfields Limited (CCL). Faced with a lack of employment options in the mine and the area more generally, Shiv and Savitha, like the majority of Karampot’s residents, eke out a living by illegally scavenging coal from the colliery and selling it on the highway. Ramesh, by contrast, is one of the minority of Santals in Karampot who are employed in Pandu Project in a regular, salaried job. Ramesh is, in fact, originally not from Karampot but a neighbouring Santal hamlet by the name of Dharutar, which had been displaced by CCL for the excavation of coal. Ramesh had been granted his job by CCL as compensation, which the company – like other public sector industries in India – aims to provide to villagers who have lost land to its projects.

Land dispossession is one of the most salient and disruptive social processes associated with extractive and other resource development projects. Local responses to it, however, are diverse, ranging from steadfast opposition (e.g. Nilsen, 2010) to compliance (e.g. Levien, 2018). Drawing on 18 months of ethnographic fieldwork^2^ in Karampot and the surrounding area, this article seeks to contribute to our understanding of the factors that shape the local politics of dispossession. It argues that, in India, there is a crucial difference, in this regard, between dispossession for private and public sector industry, which stems from the promise of compensatory public sector employment (*sarkari naukri*) that accompanies the latter. In developing this argument, the article both builds on and extends existing anthropological work on dispossessing for public sector industries in India (e.g. Parry, 2013a, 2013b, 2019; Sanchez and Strümpell, 2014). This work has notably shown that the provision of compensatory *sarkari naukri* produces class differentiation – in the Weberian sense^3^ of occupational mobility and attendant distinct lifestyles and life chances. The article supports this finding, and illustrates how the possession of *sarkari naukri* – as a life-long source of lucrative income and economic security – is at least as important an index of class inequality as the possession of land.

The implications of compensatory *sarkari naukri* for the local politics of dispossession, however, have not been adequately recognized and explored in the existing literature. The article aims to fill this gap by showing how the promise of such jobs shapes responses to dispossession in distinct ways – and, more specifically, acts to curb potentialities of resistance. In particular, the article elucidates how the provision of compensatory *sarkari naukri* does not happen automatically but requires negotiation and mediation by local leaders, who – if successful – emerge as a new kind of broker and gain particular forms of political power. Class differentiation thus goes together with *power differentiation*, and new, more centralized and hierarchized structures of power. In conjunction with class differentiation, this process – not scrutinized by other ethnographers of dispossession in India – plays an important role in inflecting local responses to dispossession. By illustrating how, in Karampot and Dharutar, these dynamics have militated against prospects of protest, the article also challenges – or seeks to adjust – Michael Levien’s (2013b, 2018: 212–36) recent hypothesis about the contexts where resistance to dispossession is more likely to arise, by highlighting the fundamental significance of compensatory formal employment in muting mobilization. This significance, moreover, bears on recent debates on class in India, and confirms – along with Parry (2021) and contra Breman (2021) – the centrality of formal employment as *the* axis of differentiation within the labouring classes.

The remainder of the article is structured as follows. The next section (‘Framing’) situates my argument within debates about dispossession and its politics. I then provide a brief historical context to Karampot and mining operations around it (‘Setting’). The following two sections (‘From protest to negotiation’ and ‘Brokering compensatory employment’) describe how the hamlet of Dharutar was displaced, focusing on the distribution of compensatory jobs, before returning to Karampot to examine some of the social and political consequences of this (‘Inequalities and patronage’, ‘Anti-mining activist or unprincipled politician’, and ‘Politics after dispossession’). I show that, while local political dynamics around dispossession have benefited some Santals, like Ramesh , they have served to curtail possibilities of mobilization for the poorest villagers, like Shiv and Savitha. The conclusion summarizes the article’s contribution and reflects more broadly on its relevance for debates about the segmentation of India’s labour force.

## Framing

There has been, going back to Marx (1977), a long history of scholarly concern with different forms of land enclosure, and its social and economic outcomes for the people who once lived off that land. In recent years, however, the increased tempo and scale of dispossession for large capital projects, and the proliferation of farmer protests against it, have attracted new levels of interest (e.g. Borras and Franco, 2012; Bush et al., 2011; Hall et al., 2011). Much of the resurgent critical literature on dispossession draws in one way or another on David Harvey’s (2004) influential theory of ‘accumulation by dispossession’ (e.g. Adnan, 2013; Gardner, 2012; Kasmir and Carbonella, 2008; Münster and Strümpell, 2014). Building on Marx, Harvey’s concept treats dispossession as an intrinsic, continuous aspect of advanced capitalism, tantamount to plunder of agrarian communities for the ever-expanding interests of capital. In Harvey’s work and closely aligned analyses, the tendency is to assume that processes of dispossession are likely to spark resistance by locals, farmers, peasants, and so on who wish to continue working their land and are thus bound to oppose its expropriation (Gardner and Gerharz, 2016; Hall, 2012, 2013). Such resistance is, moreover, often assumed in particular in contexts where tribal communities are concerned, arguably because of their strong reliance on, and cultural ties to land, as well as forests and other natural resources, as a way of life (see, for example, Padel and Das, 2010: 348–72; Shiva et al., 2011: 140–1).

However, ethnographic studies in settings of land dispossession have made this debate more complex. In the South Asian context, a review of this scholarship (e.g. Cross, 2014; Dhagamwar et al., 2003; Gardner, 2018; Levien, 2011, 2013a, 2018; Nielsen, 2018; Nielsen and Oskarsson, 2017; Parry, 1999, 2013a, 2019) makes clear that dispossession is not always met with resistance by rural people. Rather, local responses to it are considerably variegated, and involve varying forms and degrees of protest, negotiation, and compliance (Gardner and Gerharz, 2016). While in some, more publicized cases – from large dam projects in the Narmada Valley (Nilsen, 2010) to Special Economic Zones in Goa (Sampat, 2015) – significant opposition to land dispossession did materialize, in other cases dispossession took place with little or no local resistance. Dispossession for industrialization, for instance, can evoke not only fear and agitation but also hopes of modernity (Cross, 2014; Gardner, 2018), while sharp pre-existing local social inequalities can make collective mobilization difficult (Levien, 2018). Anthropologists such as Amita Baviskar (1995) and Tania Li (2014), for their part, have contested the notion of a tribal propensity to oppose land enclosure based on some kind of primordial attachment to the soil. Whereas the former has challenged the romanticized conceit of an innate tribal ‘conservationist ethic’ in relation to dispossession struggles, the latter has shown how processes of land enclosure can actually be initiated and welcomed by tribal people, as part of aspirations for a more affluent life.

The picture that emerges is one of significant variation in political responses to dispossession, from fierce resistance to acquiescence and even welcoming it. The complex challenge that then arises is to identify the factors that affect and explain local political reactions to dispossession. Especially useful, in this context, is Levien’s call for a ‘comparative sociology of dispossession’ (2018: 4), which examines empirically how distinct, specific forms (or ‘regimes’) of dispossession ‘interact with diverse agrarian milieux’ with particular economic foundations, social structures, and political histories to generate particular local political responses (Levien, 2018: 4). In this vein, the article presents an ethnographic analysis of the interaction between India’s state-run mining industry and an Adivasi community in Jharkhand. Levien’s and other recent work on land acquisition in the South Asian context (e.g. Bedi, 2013; Cross, 2014; Jenkins et al., 2014) has tended to focus on (state-assisted) dispossession for private capital such as Special Economic Zones. This article, on the other hand, is about dispossession for a public sector industry in India, which in Jharkhand’s coal belt has constituted the more common form of land expropriation.

Authors such as Levien (2011, 2018: 31–62) and Parry (2013a) have considered the difference between dispossession for private and public sector undertakings in India mainly in terms of the distinct ideologies that underpin each type of dispossession. Dispossession for public sector projects is associated mainly with India’s earlier, developmental Nehruvian programme and its social commitments, where land was acquired for projects serving a ‘public purpose’. Dispossession for private capital projects, on the other hand, is undergirded by a neoliberal logic whereby the state acquires land ‘for any private purpose representing “growth”’ (Levien, 2018: 26). But for the politics of dispossession, this article suggests, much more significant is the promise of compensatory *sarkari naukri* as a distinguishing feature of dispossession for public sector industry.^4^ Such employment, the privileges that come with it, and the ways in which it affects one’s class position play a decisive role in shaping political responses to dispossession. As I discuss in the concluding section, this proposition also bears relevance for debates about India’s wider class structure, and points to the prominence of formal employment – as opposed to any form of work in the informal economy – as the main qualitative line of division within the labour force (Breman, 2021; Parry, 2021)

More specifically, the article illustrates how compensatory *sarkari naukri* for land loss led to processes that have stifled prospects of protest – precisely in a context in which, according to Levien (2013b, 2018: 212–36), resistance to dispossession could have been considered likelier to emerge. Drawing on his and other studies on dispossession, Levien goes on to propose that opposition to it may be more probable where agriculture dependence and profitability are higher, pre-existing inequalities more subdued, and histories of peasant activism stronger. While Karampot and Dharutar fulfil the first condition only partially – agriculture is relied on for subsistence but is not profitable – they score high on the second and third. Compared to the rest of Indian society, Adivasi communities across central and eastern India are known for their relatively egalitarian structures and values (Shah, 2018), and, moreover, have long histories of political agitation against land expropriation by state and capital (Bates and Shah, 2017). This in particular has been used to explain resistance to dispossession by Adivasi communities (Levien, 2013b: 375; Parry and Strümpell, 2008: 54). The article, however, shows how compensatory jobs for dispossession – of the kind provided only by state-owned industries – had two particular effects that have acted to mute possibilities of resistance, effectively overriding those factors that have been argued to facilitate it. The first is intra-community class differentiation – between those with and without compensatory jobs – where the article’s findings are in line with other studies of dispossessing public sector plants in India (Parry, 2019; Parry and Strümpell, 2008; Sanchez and Strümpell, 2014). The second, related effect is one that these analyses have largely not drawn attention to, namely power differentiation – whereby those who broker access to compensatory jobs gain consolidated forms of political power.

## Setting

The state of Jharkhand lies in the plateau region of Chotanagpur, abundant in natural resources and home to a range of tribal groups. As in other Adivasi villages not subject to the sharp caste hierarchies that characterize the Indian plains (Shah et al., 2018: 27), Adivasis in Karampot and the area historically reproduced themselves under generally more egalitarian conditions: while economic differences between households were not entirely absent, they were relatively minimal and transitory. Coal mining operations in the Karampot area commenced in the 1970s, when a number of private underground collieries were established around the village by higher-caste Hindus, and attracted Santals and other locals to work as coal cutters. In 1973, acknowledging the importance of coal as a national asset, the mining industry was nationalized, and these private mines – like others in the country – were gradually either shut down or brought under state ownership. The next few years would see the construction of new, state-owned opencast mines in the area under the management of CCL – a subsidiary of Coal India Limited (CIL), the national corporation that now operates the vast majority of collieries of the country.

One of these new mines was Pandu Project, which began construction in 1982 just a few kilometres from Karampot. To make way for it, the government had in 1964 acquired more than 3,000 acres of land from seven villages.^5^ In addition to forest and grazing land surrounding Karampot, land acquisition included the entire tract of Dharutar, the neighbouring Santal hamlet. When the building of the project began, Dharutar households had to surrender agricultural land, and for this 39 of the village’s 49 households at the time received a compensatory job from the company. The offer of such jobs is one of the most prominent distinguishing features of dispossession for public sector projects in India vis-à-vis dispossession for private capital. While dispossessing private projects may offer different forms of material compensation for expropriated land (Levien, 2018) and/or casual wage labour opportunities (Gardner, 2018), state-owned industrial projects in India generally carry a policy – rooted in the country’s developmentalist, Nehruvian era – of providing *naukri* (formal employment) to villagers dispossessed of a certain minimum amount of land.^6^ Nehruvian public sector industrialization centred not only on growth but also employment generation (Fernandes, 2007; Parry, 2003); as part of this vision, CCL offered to employ one Santal from each Dharutar household who had lost at least 3 acres of private land. Consequently, in most Dharutar households one member received a *naukri* in the new project. It is important to underline from the outset the value of *naukri* in the Indian context, and its superiority to virtually any conceivable form of monetary compensation for dispossession. Jonathan Parry (2013a, 2019) has asserted that the main divide within India’s labouring classes is between the 92 per cent engaged in precarious, informal work, and the exclusive 8 per cent who have *naukri* (Harriss-White, 2010; Nath, 2008) – which, crucially, provides long-term economic security. Moreover, within the category of formal employment, *sarkari naukri* – public sector jobs, of the kind CCL provides – is widely regarded as the most remunerative and protected (Parry, 2013a, 2019).

While jobs for dispossessed private land were attained by Dharutar villagers in 1982, the village was not in fact displaced until 2008. In that year, it was announced that a new mining site would be excavated on Dharutar’s remaining territory, which comprised the village’s houses as well as patches of common land. The displacement of the village – which by now had grown to 118 households – was consequently initiated, and proceeded over the next couple of years. Subsequently, a second round of compensatory job distribution took place in 2010, for the village’s common land plots, which various households had been using informally for cultivation or housing. This disbursement of jobs, however, was very different from the first, and involved, as I describe in the next section, close intervention by Santal politicians from the locally dominant Jharkhand Liberation Front party, or JMM.

The JMM has a distinguished history of resistance in the Jharkhand region. In the 1970s and 1980s, much of the region had been swept up by the protest activities of the party – then an activist movement that had spearheaded a militant protest campaign against land alienation and exploitation of Adivasis by Hindu ‘outsiders’, and for the establishment of a separate Jharkhand state (Devalle, 1992). The JMM’s combative campaign had mobilized and gained widespread support among Adivasis and become seared into popular memory. Importantly, the JMM had been not only pro- Adivasi but also anti-mining: its agrarian protest had famously combined with a trade unionist struggle against coal companies around compensation to displaced peasants (Devalle, 1992). Both displacement and mining, then, had been main sites of JMM activity, and its members are considered Adivasi activists on both these fronts. In what follows, however, I look at how an ostensible JMM anti-mining activist turned to negotiating with CCL to broker compensatory jobs, and at the social and political consequences this has had in Karampot.

## From protest to negotiation

In late 2007, notifications were hung on the doors of Dharutar’s houses, informing villagers about the hamlet’s upcoming displacement. At first, this generated anger and protest. Its main organizer was Budhram Manjhi – a Santal from the village who was a political worker for the JMM. Budhram had come from a locally dominant Santal family that had had a history of engagement in different types of local brokerage: his grandfather had served as the village tax collector for the local Hindu *raja*, and his father had worked for a local Hindu moneylender. Budhram himself became exposed to the JMM at a young age, in the 1980s. Keen to explore life outside Dharutar, he began frequenting the party’s vigorous demonstrations and rallies. Increasingly drawn to the movement, Budhram eventually became a political worker for Chitu Besra, a rising JMM Santal politician who hailed from a village near Dharutar and in 2003 became JMM’s General Secretary. Deployed on various tasks in local government offices, Budhram got to circulate among other, more experienced political workers, build his own network, and gain the practical know-how for getting things done. Using his connections, he started acting as a broker between villagers and local state bureaucracy, assisting the former with obtaining caste certificates or gaining access to state welfare schemes. When the expansion of Pandu Project was announced, Budhram – with the support of Chitu – mobilized villagers to protest against Dharutar’s displacement. Recalling that time, Ramesh and other interlocutors from Dharutar described demonstrations in front of Pandu Project’s offices, demanding that the colliery should be shut down.

This incipient opposition movement, however, was short-lived. Budhram and Chitu soon came to the conclusion that foiling the mine’s expansion through protest was unlikely to succeed – not least because CCL had already acquired the tract of land long ago, in 1964, and recompensed villagers for loss of agricultural land in 1982. Instead of fighting against the inevitable, they reckoned, it would be better to seek to obtain more jobs from CCL for the community – which, as I detail below, was not as straightforward as in 1982 this time around. CCL, for its part, was also more inclined to engage with Budhram and Chitu than face intractable protests by villagers. This was arguably especially critical for Pandu Project’s management, given the strong political history of the region, and particularly the JMM, to which Budhram and Chitu belonged. Wishing to avoid agitation, then, Pandu Project’s management opened its doors to Budhram, which in turn led to the waning of the nascent protest movement against the colliery. The character of the political struggle transformed: it was now no longer about the actual loss of land, but about compensatory employment in the form of *sarkari naukri*. In many ways, the effort to obtain these jobs was consonant with villagers’ own aspirations. Especially for younger Santals, the expansion of the colliery did not spell the destruction of a cherished rural way of life. Instead, it represented a unique opportunity to escape the unreliable combination of subsistence agriculture under demographic pressure and casual wage labour, and to advance hopes of economic security through the prospect of a prized *naukri*. Indeed, as Parry has summarized, ‘The crucial thing that all those with *naukri* – especially *sarkari naukri –* share is job security, which is to say a relatively predictable future’ (2019: 65). This is, importantly, as much the case for a mechanical assistant as for the project manager.

Since the first round of dispossession in Dharutar, in 1982, the number of households in the village had increased considerably, and many younger Santals from the new households were now eager to gain CCL employment in exchange for land. As *sarkari naukri*, a CCL job is especially desirable, and losing land is seen as the only way to attain it. Gaining CCL employment or any other public sector job via the standard recruitment route – and despite affirmative action measures for Adivasis – was not even a remote possibility for most Santals. Not only does it require skills and credentials that they mostly lack but it also hinges on, it is commonly believed, connections and bribery.

There were, however, two substantial hindrances that stood in the way of younger Santals’ hopes for employment. First, compensation by CCL is only granted for the dispossession of formally owned tenancy land. Conversely, what is known deedless land – which Adivasi households have often been using informally for decades – is not recognized by CCL for compensation purposes. As it happened, all the tenancy land in Dharutar had already been compensated for by CCL in 1982; it was for this land that some of the older Santals in the village had obtained their compensatory jobs back then. The land that was now about to be expropriated, by contrast, was all deedless, which as such did not make its cultivators eligible for employment.

The second obstacle had to do with broader changes in the coal industry that had taken place since 1982. Roughly up until the mid-1990s, it had been customary for CCL to compensate for loss of land through jobs as part of the developmentalist objective of absorbing labour into industrial public sector employment. Thereafter, however, the implementation of the company’s jobs-for-land policy has become increasingly diluted, as a result of a sharp reduction in the number of available company jobs. This downward employment trend ought to be situated within the context of India’s economic liberalization in the early 1990s. While, prior to liberalization, many of India’s public sector enterprises – including CIL – had played an important role in boosting both output and employment, they had done so with excess manpower and at the cost of efficiency and profit (Dhananjayan and Shanti, 2007), which had been tolerated for the sake of generating employment. With liberalization, however, profit maximization took precedence and dictated significant downsizing of the regular workforce in state-owned enterprises (Bear, 2013; Dhananjayan and Shanti, 2007; Parry, 2003, 2013a). This, in turn, has resulted in a considerable decline in the number of available jobs in the mining industry – especially for less-skilled labourers – and constrained CIL’s ability to offer jobs as compensation for land loss (Fernandes, 2007; Herbert and Lahiri-Dutt, 2014), as the company itself has conceded:

In the past, subsidiaries found it relatively easy to acquire land, if they were able to offer jobs. Partly because of this practice, subsidiaries have built up their labour force beyond their needs. This has contributed to the heavy losses many mines incur and eroded the competitiveness of the coal industry. The subsidiaries may still need to hire people in selected locations and continue to give preference to those whose livelihood will be affected by coal mining operations. However, increasingly subsidiaries will need to develop other ways and means to compensate landowners and others adversely affected by their projects. (CIL, 2008: 1)

In line with this, and in an effort to reduce the number of compensatory jobs distributed, CIL has encouraged subsidiaries to offer increased monetary compensation for dispossessed land in lieu of employment (Business Standard, 2013; Hindu Business Line, 2018). Indeed, different studies have shown that CIL’s jobs-for-land policy has in recent years been poorly implemented, with the company unable or unwilling to provide employment positions to dispossessed villagers who, in principle, should have qualified for them (e.g. Ahmad and Lahiri-Dutt, 2014; Jain and Bala, 2006). One of the better-documented cases comes from CCL’s Parej East Project – just under 10 miles from Karampot – where, following workforce cuts, the offer of *naukri* to dispossessed villagers had by the late 1990s effectively ceased to be an option (Herbert and Lahiri-Dutt, 2014).

## Brokering compensatory employment

Despite the hurdles around compensatory employment, Budhram was determined. The only way for Dharutar villagers, he figured – including his own household – to have a chance at CCL employment at this point was to first of all have their deedless land formally registered. The key, he realized, lay in a specific clause in CCL’s policy document. Pertaining to ‘tribals cultivating land under traditional rights’, the clause makes a provision for authentication of ownership as a means to claim compensation for that land’s acquisition. Authenticating land, however – and then negotiating employment – is a complex process, which requires interacting with both government and CCL officials. Budhram, who as a political worker had some experience in this domain, took upon himself the task of intermediating between villagers and these officials to try to deliver more jobs to villagers. This endeavour, moreover, also fed into Budhram’s own political ambitions: here was an opportunity to expand his brokerage activities, prove his own value and power, and make the transition into becoming a *neta*, or political leader, in both the community and the party.

There remained, however, a financial issue. Registering land entails the production of a litany of official documents and signatures which, as Budhram explained, requires greasing the palms of different government officers. Land authentication thus came with an informal fee, which Budhram intended to collect from villagers: around 50,000 rupees – roughly equivalent to 150 days of coal peddling – for the titling of a plot that would make a household eligible for a compensatory job. Some villagers, like Ramesh, had a father or uncle who was already a CCL employee – from the distribution of jobs in 1982 – and could help them with this substantial sum. Others used savings from years of coal peddling and wage labour.

Within a couple of years, and after numerous visits to local government offices, Budhram efficiently succeeded in registering the land plots of those villagers who had provided him with the necessary payment. Next, he set out to present the land papers to CCL and, using the threat of further protest, press management to find posts in the project for all newly minted Santal land owners. In total, 62 jobs were provided to Santals from Dharutar between 2012 and 2014 – including to Budhram and each of his two sons – mostly as assistants in the colliery’s machinery workshop. Most of the newly employed Santal households have taken up residence in the CCL housing colony not far from the project; the rest – including Ramesh’s and Budhram’s – have relocated to Karampot, where they erected large cement houses and make up a privileged minority of only about 10 per cent of the village’s 146 households. In our conversations, virtually all of them acknowledged that without Budhram their employment would not have been possible.

Most Santal households from Dharutar were thus able to gain employment in the project following displacement. A minority, however, were not. Of the 69 households that had been added to Dharutar between 1982 and 2010, about a dozen were left without jobs: they had not provided Budhram with the money to title their land, and consequently were left out of employment negotiations. While Budhram attributes this to these villagers’ lack of trust in him, they insist that they simply did not have the means to pay for the registration of land. Unlike Ramesh, they did not have relatives with CCL jobs to help, and did not manage to arrange the money in time. These non-employed households – who in addition have been stripped of their plots of land – are in fact very much disgruntled with Budhram, who they feel has deprived them of the jobs they were supposed to get.

And yet, given the paucity of public sector employment in India and developments within CIL in particular, the relatively large number of jobs that were handed out to displaced Santals from Dharutar is striking. When compared to cases such as in Parej East (Herbert and Lahiri-Dutt, 2014), and based on my own inquires in other projects in the area, it appears that the implementation of CIL’s compensation policy can vary considerably between sites. While its distinct localized outcomes arguably depend on a wider range of factors than can be covered here, my findings suggest that salient among these – especially in light of CIL’s increasing reluctance to provide jobs – is the form and degree of negotiation, political pressure, and brokerage applied by local leaders. As a higher-level employee in Pandu Project told me, ‘Nowadays, company officials do not give away jobs voluntarily. Unless you know exactly what villagers are entitled to, unless you have the skills to insist on jobs, officials will try to sell you other types of compensation.’ In Dharutar, Budhram’s navigation of the compensation scheme and state bureaucracy – with the backing of Chitu and history of JMM protest – played an essential role in extracting more favourable outcomes from displacement.

However, as I show in the remainder of the article, the relatively successful disbursement of CCL jobs, as facilitated by Budhram, has been a double-edged sword. On the one hand, it secured the livelihoods of a significant section of displaced Santals. But on the other, it has been accompanied by two countervailing processes, which I describe next. First, public sector employment has created new forms of differentiation between employed and non-employed Santals. Second, Budhram’s intermediation of coal company jobs has consolidated his political power, and produced a more centralized, hierarchical local political structure. Together, these two processes have acted to curtail prospects of mobilization by and for poorer, non-employed villagers.

## Inequalities and patronage

In Karampot, there are now glaring differences between CCL-employed households, originally from Dharutar, and native coal-peddling households, who have not lost their own agricultural land to the mine and thus could not get jobs. While Shiv, for instance, lives in a mud house and travels on foot and by bicycle, Ramesh resides in a large concrete house and owns a motorcycle. While Shiv’s young daughter goes to the local, government school, Ramesh sends his to a private school outside the village. While Shiv, in the evening, usually drinks traditional, homemade beer and wine, Ramesh often opts for bottled whiskey and rum. And while the celebration of Shiv’s marriage to Savitha, several years ago, was a modest village affair, employed Santals like Ramesh now throw much more opulent wedding parties, with decorated marquees and catering. The adoption of such practices and consumption patterns by employed Santals, enabled through their relatively substantial salaries, reflects middle-class aspirations as well as emulation of higher-caste norms and ways of life (see also Higham and Shah, 2013). This, as I discuss in more detail elsewhere (Noy, 2020), has created new class differentiation in Karampot, between the minority of employed households and all the rest. Compared to the visible class mobility of the former, the latter feel increasingly excluded and marginalized. This new differentiation, I should stress, is not simply an intensified version of the low levels of disparity that had existed in Karampot before the arrival of mining. Rather, state employment has produced class differentiation – that is, inequality of a different qualitative order. *Sarkari naukri* has brought employed Santals into the privileged stratum of formal, public sector workers – firmly separated from all other, precarious workers (Parry, 2019: 39–75).

Budhram himself is arguably the primary example of the upwardly mobile lifestyle of Santal CCL employees. He owns not only a motorcycle but also an SUV; he is the only person in Karampot whose house has not only a Western-style toilet but also a shower; and his wife, in higher-caste fashion, is disengaged from any type of extra-household work and is rarely seen outside the family’s gated compound. Differently from other employed Santals, though, Budhram harbours not only middle-class but also political aspirations, and has used his brokerage of employment to fashion himself as a benevolent patron and *neta*. No one else around has delivered ‘*these* kinds of benefits’ to Santals, Budhram proudly told me, and it is from this that he now draws his legitimacy.

Budhram’s brokerage of CCL jobs has enhanced and entrenched his position as a local leader and patron. Already, prior to the colliery’s expansion, as I described, Budhram had acted as a broker between villagers and local state bureaucracy. Now, however, he has asserted himself as the interface between villagers and CCL – the national coal corporation and purveyor of *sarkari naukri*. While Budhram’s brokerage of CCL resources has built on, and cannot be separated from, his previous brokerage activities, it nevertheless operates on a different scale. Helping villagers with various administrative tasks and local welfare schemes is one thing; delivering *sarkari naukri* – the most sought-after form of employment – quite another. This scaling-up has considerably boosted Budhram’s influence and power.

A new scenario of patronage has emerged around the ability to negotiate compensatory jobs and, more generally, any form of interaction with the project. This has placed Budhram in a genuinely influential position not only vis-à-vis those who already have jobs – who now for example seek his assistance in gaining work promotions – but also others in Karampot, who have not yet lost any land of their own but, following the events in Dharutar, view Budhram as the conduit to employment *in case* of dispossession. Indeed, Shiv and Karampot’s other coal peddlers believe that their village too will eventually be submerged by the expanding colliery, and many now think about their land in terms of what it could yield when expropriated. This makes it sensible for them to remain in favour with Budhram, in order to maintain chances of potential future employment.

Budhram’s key role in delivering CCL employment has cemented his status not only in the community but also in the JMM as a capable political worker, and has proven a pivotal step in his political ascent. Since Dharutar’s displacement, Budhram has been elected a JMM committee member and, no less significantly, secretary of the party-supported regional labour union for mine workers, the Jharkhand Colliery Mazdoor Union (JCMU). Budhram was thus able to parlay his engagement with CCL and its compensation scheme into own political advancement, thereby gaining political mileage out of dispossession. Budhram seems to now have considerable bargaining power vis-à-vis CCL, as was also confirmed to me by leaders of other unions in the area. While the JCMU is not a national union, they explained, locally it has become dominant – not least due to its affiliation with JMM. In Pandu Project, for example, of all unions present the JCMU has the largest number of members. With the initial protest movement against the project, then, Budhram had already demonstrated to CCL his ability to mobilize villagers and generate opposition; his formal position as the JCMU secretary has now only augmented his political power. In theory, as company officers, too, agreed, Budhram and the JMM have the means to disrupt local coal production to a substantial degree. The threat of strike is, indeed, one often used by Budhram as a bargaining chip with CCL. In practice, though, strikes virtually never take place, as I discuss later on. To local Santals, this is hardly surprising: while on the surface Budhram might appear as an Adivasi activist, in Karampot, as I show next, many villagers think of him very differently – not as an activist but an unscrupulous broker-politician, who has used the relationship he forged with CCL mainly for his own benefit.

## Anti-mining activist or unprincipled politician?

Those Santals from Dharutar who have been left without CCL jobs – and who now reside in either Karampot or a cluster of crumbling mud houses next to the mine – have quite a different version of events to share of the village’s displacement than the one told by Budhram. The reason they did not gain employment, these Santals claim, is because they are not close with Budhram: jobs were arranged first and foremost for Budhram’s various kinsmen in Dharutar, and then for others who had the means to pay for land registration – even if they were not from the village. In this, they are not entirely wrong: Budhram does admit, when directly asked, that since there was in fact more deedless land to register than villagers could pay for, in a number of cases he accepted money from people from outside Dharutar, who thereby effectively bought CCL jobs for themselves through him. I know personally of at least one Santal in Karampot who is neither originally from Dharutar nor had any land there, but who managed to attain a CCL job in this way.

There is, at any rate, a strong sense among jobless displaced Santals that Budhram assisted primarily those in his inner social circle. Those outside of it, they say, were not always told about the meetings in which Budhram informed villagers about land registration; learned about the cost of land registration only at the very last minute; and consequently were not able to arrange the money for this in time. These jobless villagers point out that the lion’s share of jobs have gone to people within Budhram’s extended kinship network, while Budhram’s own household has the largest number of jobs of all. Such claims about corruption in the distribution of employment positions are difficult to either verify or refute. They do, however, reflect a view about the informal, untransparent, and inequitable nature of the kind of brokerage and patronage politics enacted by Budhram as well as other *netas* in the area.

Indeed, belief in the immoral conduct of *netas* extends well beyond the group of jobless displaced Santals from Dharutar, and is prevalent among Karampot’s coal peddlers. While they still retain their small agricultural plots, these villagers too have grievances about the colliery’s toll on their living environment: from the pollution that contaminates the air and soil, to the blasting that cracks the walls of their mud houses, to the enclosure of grazing and forest areas around the village. For all this, they have received nothing in return. Because they have not been *directly* dispossessed, they are not eligible for employment, and otherwise have been offered no economic opportunities through the colliery, nor any local services or benefits such as paved village roads or a better school. Instead, apart from stealing coal to peddle, villagers like Shiv and Savitha only have access to casual wage labour loading coal onto trucks in the mine’s depot yard, which is what CCL provides to ‘project-affected persons’ around the colliery. Talking about their predicament, Shiv and Savitha point a finger at Budhram. Since the expansion of the mine, they feel, instead of working on behalf of poor villagers Budhram has been concerned mostly with his own political advancement. ‘Even at the time of the protest in Dharutar’, Shiv said, ‘Budhram and Chitu were just using the demonstrations to show their power. They shouted slogans into the microphone but in the end went into the project office, closed the door, and made all kinds of deals.… *Netas* care not about people like us but their own position.’

Santal *netas* such as Budhram tend to speak of their actions as a charitable service to the community. While various non-Adivasi fixers, they say, only try to cheat Santal villagers, *netas* like themselves are committed to supporting their tribesmen. In this, Budhram evokes the notion of *seva*, or disinterested service. For many of my interlocutors, however, Budhram’s conduct is associated not with *seva* but with *netagiri*, or self-interested politics. While *netas* like Budhram may have started off as ‘community workers’, as they climb up the political ladder invariably they become corrupt (see also Mayer, 1981; Ruud, 2001). In Karampot, this local perception of Santal *netas* has translated into widespread disillusionment and cynicism about politics. Indeed, the rise of *netas* like Budhram, and the corrupt conduct associated with them, has not gone morally uncontested. This is best illustrated by the case of Siamlal, Shiv’s uncle and one of my older, closer interlocutors in the village. Like Budhram, Siamlal was, in his younger days, actively involved in the JMM protest movement. But unlike Budhram, Siamlal has ultimately chosen to veer away from the immoral world of politics. Increasingly frustrated with the path taken by the JMM and its *netas* – from Budhram to higher-level leaders – Siamlal has turned to a life of religious piety, and spends most of his time in the small Hindu temple across the highway from Karampot.

Budhram’s material and political self-seeking, for Siamlal, is only a symptom of a much larger problem. Corrupt regional and national JMM leaders, too, have failed to fulfil the promise of bettering the situation of Adivasis, which was at the centre of their political campaign prior to Jharkhand’s independence. Such a view fits within a narrative of corruption that has developed around the JMM since the 1990s, when the party was involved in a number of high-profile bribery scandals. While the JMM, Siamlal believes, began as a grassroots, activist movement, as it gained influence it has become tainted, and morphed into a clique of self-interested politicians. Disaffected with this reality, Siamlal withdrew all engagement with the party a few years after Jharkhand’s independence. ‘People had been promised work and development’, he said, ‘but none of this happened, because party leaders only care about themselves.… If *they* are behaving like this, eating up huge amounts of public money, what can you expect from local *netas* like Budhram?’

Siamlal’s retreat from politics was paralleled by an embrace of a more ascetic lifestyle. Gradually increasing his visits to the temple across the road, he eventually took over from its elderly priest, and now dedicates his time to maintaining the shrine and conducting the daily rituals. ‘Adivasis are still being exploited’, Siamlal declared in one of our conversations in the temple – not only by CCL, which is taking away their land and harming their environment, but now also by *netas* like Budhram who, instead of mobilizing villagers like the JMM used to, are being co-opted by CCL and are preoccupied mostly with their own position. Adivasi values of egalitarianism, Siamlal and other older Santals feel, are being increasingly eroded – not only through the immorality of Santal *netas* but also the conspicuous display of wealth by newly employed Santals. From a community in which everybody used to live on roughly the same terms, they lament, Karampot has become a place where some people now drive around on flashy motorcycles and in SUVs.

Juxtaposed with Budhram’s life trajectory, Simalal’s asceticism represents an alternative moral world, and serves as an ethical commentary on the political economy of self-interest and differentiation that has been enfolding the community. While Siamlal’s views echo those of many others in Karampot, especially from the older generation, his decision to effectively renounce this political economy is nevertheless unusual. While many villagers are critical of Budhram in private, they feel they have no choice but to rely on him for their interaction – present or future – with both CCL and state bureaucracy. As I show next, Shiv, Savitha and other coal peddlers believe that the community is too divided, and Budhram too influential, for them to initiate any political action on their own.

## Politics after dispossession

Despite the discontent among Karampot’s coal-peddling Santals, politics around Pandu Project has continued to be characterized by relative quiescence rather than protest. Truck-loading wage labour is a case in point. As I have described, this casual, informal work is what CCL aims to provide to ‘project-affected persons’ who do not qualify for employment. To make matters worse, in Pandu Project the supply of truck-loading work, according to my interlocutors, has only been diminishing. It is now scarce and highly irregular, which is a source of widespread indignation in Karampot. In other areas in the district, similar anger about truck-loading work has been reported to lead to large demonstrations. In Karampot, there has been nothing of this sort. When I asked Shiv, Siamlal and others why villagers do not start an *andolan* (protest movement), they replied that there is not enough unity in the community. ‘Some people now have CCL jobs,’ Siamlal said, ‘so problems with truck loading don’t bother them.’ This statement crystallizes the corrosive effect of class differentiation on political solidarity (see also Parry, 2013b). New inequalities between employed and non-employed Santals – generated through dispossession and compensation, with Budhram’s mediation – have undermined the commonality of interests, and consequently the ground for collective action. CCL-employed Santals now have other concerns than coal peddlers; while they might sympathize with their plight, they are unlikely to partake in any agitation against the project that might jeopardize their own jobs.

But many coal peddlers also explicitly referred to Budhram as a reason for the lack of protest, highlighting his relationship with CCL. Take, for example, the possibility of strikes in the project, which in theory could be a powerful tool to press demands for more truck-loading work or other benefits for non-employed villagers. As I witnessed on various occasions, the threat of strike is one often floated by Budhram in meetings with Pandu Project officials. Beyond this, however, strikes never seem to materialize. Indeed, CCL officers confirmed to me that since the project’s expansion – and the short-lived opposition movement in 2008 – there has been no attempt at industrial action in the colliery. ‘*Netas* like Budhram’, they said, ‘wouldn’t go so far as to disturb coal production in any serious way. Budhram himself is a CCL employee and, in case of a major disruption, wouldn’t receive his salary!’ There seems to me, however, to be a deeper underpinning to this dynamic. On the one hand, Budhram’s political power, and the history of JMM resistance, means that his threats of strikes carry real potential weight. But on the other hand, much of Budhram’s current local sway has been derived precisely from his engagement with CCL and brokerage of company jobs; over-agitating against the project would mean putting this at risk.

Budhram, for his part, argues that the amount of truck-loading work hinges on production levels in the quarry, beyond his control. But coal-peddling Santals are not convinced. From their point of view, as a *neta* Budhram has not done enough to address their situation, having become too closely entangled with CCL and too swept up in party politics. At the same time, however, coal peddlers like Shiv and Savitha do not believe they have the capacity to arrange any kind of protest outside of Budhram’s purview. They referred to themselves, in this context, as ‘simple’ and ‘small’, without the power to start an *andolan* on their own. Such sentiments, I propose, have to be understood in relation to Budhram’s cemented local political grip in the wake of dispossession, which has left poor Santals feeling increasingly politically disempowered.

A couple of years ago, for example, Savitha and a group of women from the village plucked up their courage and went to the Pandu Project office, with the intention of voicing their dissatisfaction with the scarcity of truck-loading work. There, they encountered Budhram, who had just come out of a meeting with the project manager. Budhram, Savitha recounted, told her and the other women off for trying to approach CCL officers directly and to intervene in matters they ‘don’t understand’, and ordered them to go back to the village. Instances such as this are taken by my interlocutors to indicate that any political action they might initiate would only be taken over by the *netas*. Santal villagers, it seems, have not only become increasingly dependent on *netas* like Budhram but also increasingly entrapped by them; any attempt at grassroots political activity outside their purview is essentially perceived as futile. That a good number of villagers are themselves CCL employees – and thus closer to Budhram in terms of class than to non-employed locals – only exacerbates the inability to instigate protest that circumvents him (cf. Raj, 2019).

Given the rich past and historical accounts of vigorous bottom-up Santal activism (Devalle, 1992), this prevalent sense of popular political incapacity was striking. On one level, it is embedded within Santal villagers’ broader disillusionment with the JMM and its corrupt politics. But on another level, I argue, it is also the result of the effective monopolization of localized political power by Budhram, as part of a new class of Adivasi *netas* with new forms of political authority. The accumulation of particular forms of political power through the arrival of the colliery has allowed Budhram to establish exclusive political dominance, partly in reality and perhaps more so in villagers’ perception. Indeed, for many of them it is difficult even to imagine possibilities of collective action independent of, or circumventing, *netas* like him. ‘If you try to raise a voice about something,’ non-employed Santals said to me – for example, the shortage of truck-loading work – ‘it will only go up to Budhram, and the matter will probably end there.’ In their discussion of what they call a ‘politics of resignation’, Benson and Kirsch (2010) describe a similar feeling of political paralysis that, in their analysis, stems from the devious tactics employed by mining and other corporations to rebut critique and sustain operations. In Karampot, however, people’s sense of political resignation is experienced most directly in relation not to CCL but Santal *netas* and their politics around mining. Rather than an agent of grassroots politics, Budhram – as a type of Adivasi broker-politician with consolidated power – has come to be perceived locally as a strain on it, and an impediment to political action by and for poorer, non-employed Santals.

## Conclusion

Ethnographic analyses of encounters between rural communities and land-acquiring projects have revealed the diversity of local reactions to dispossession, from overt opposition to ready compliance. The challenge that then emerges is to trace the different factors and conditions that give rise to particular responses to dispossession. While this endeavour would require further, comparative ethnographic research, this article has drawn attention, in the Indian context, to the distinction between dispossession for private capital and for public sector industry as a critical parameter. It is, crucially, only the latter that can provide *sarkari naukri* as compensation, which in Karampot/Dharutar had a prominent impact on the political response to dispossession for mining.

Ethnographically, the article has considered the role of a local Adivasi political leader in negotiating and mediating the distribution of *sarkari naukri* to dispossessed villagers, and the consequences of this. Crucial here is the value of *sarkari naukri* as an enabler of security and class mobility – especially at a time in which, owing to the dictates of economic liberalization, access to public sector employment has become significantly more restricted. On the one hand, the political pressure and brokerage applied by Budhram was successful in delivering *sarkari naukri* to a relatively large number of displaced Santals, which has allowed them to achieve a considerable degree of class mobility by joining the privileged ranks of public sector employees. Indeed, thinking in terms of accumulation by dispossession, dispossession for public sector industry in India generates – depending on local negotiation and pressure – accumulation for the labour aristocracy of *sarkari naukri*, or, more precisely, for those villagers who, as compensation recipients, are brought into its fold. In Karampot, this has spurred two particular processes. The first is class differentiation, which has also been described by other authors in similar contexts (Parry, 2019; Parry and Strümpell, 2008; Sanchez and Strümpell, 2014). This differentiation has not only enhanced poorer Santals’ sense of marginalization, but has also chipped away at the kind of solidarities that can be conducive for collective action (see also Levien, 2018: 179–211). The second, accompanying process is that of power differentiation, far less scrutinized by these authors. Through his brokerage activities, Budhram emerged as the local conduit to compensatory *naukri*, which has consolidated his patronage and political power. Budhram is revealing of a particular type of such power, combining *sarkari naukri* (which Budhram himself has) and links to political parties (the JMM) and trade unions (the JCMU). He is part of a political class of broker-politicians representative of those with *sakari naukri*, who make up a new local political elite. In parallel, Budhram’s working relationship with CCL, and his entry into the political system as a new *neta*, have in the eyes of local Santals tainted him and eroded his commitment to the community. Together, Budhram’s enhanced political grip, and the view of him as corrupt, have driven poorer, non-employed villagers into a state of heightened political cynicism and resignation.

The conjuncture of class and power differentiation – both related, directly or indirectly, to the offer of *naukri* as part of the terms of compensation for land loss – has acted as a hindrance to potentially more comprehensive and radical types of protest, which have been documented in other rural settings of dispossession in India (see Levien, 2013b). In and around Karampot, such protest could encompass, for instance, project-affected Santals left out of any colliery compensatory benefits, and challenge the uneven development trajectory that ventures like Pandu Project set off. While both class disparities and Budhram’s conduct have not been impervious to moral critique, which draws on Adivasi values of egalitarianism, the local divisions and more rigid political hierarchy that emerged in the wake of dispossession and compensation have made it more difficult to translate this critique into political action. Indeed, in Karampot, class and power differentiation have effectively outweighed other features that have recently been suggested to be likelier to foster resistance (Levien, 2018): a pre-existing relatively egalitarian structure (compared to rural caste society) and a vigorous history of protest (through the JMM movement in the 1970s and 1980s, in which Budhram himself had been involved).

These dynamics evince the importance, in the Indian context, of compensatory formal employment and concomitant class mobility – limited to land loss for public sector industry – as a salient variable in shaping local politics around dispossession. This, in turn, has implications for wider debates about class in India. In a recent exchange, Breman (2021) and Parry (2021) crystallized two diverging positions on the segmentation of India’s workforce. Challenging Parry’s assertion about the distinctiveness of the *naukri*–informal work divide within the labouring classes, Breman contends that labour is split up not ‘in a two-class dichotomy’ but in multiple ‘demarcated zones’, characterized by different forms of work combined with ‘greater or lesser shades of vulnerability’ (2021: 143–7). This article’s findings, however, about how *naukri* effectively inhibits resistance to dispossession, lend support to Parry’s claim rather than Breman’s – and imply that *naukri* is indeed the most crucial axis of differentiation within the workforce, qualitatively different from all other distinctions within it.

## Notes

1 Most names of places and personal names have been changed.

2 The research on which this article is based was carried out between 2015 and 2017. Methods used included a comprehensive household survey in Karampot, in which the livelihood activities of each member were recorded; numerous in-depth, semi-structured interviews, and more informal, open-ended conversations with different villagers (both coal peddlers and colliery employees), project officers, local political workers and leaders, and labour union leaders; and participant observation in different villagers’ everyday lives and work.

3 As Parry notes, the Weberian notion of class usefully allows for important distinctions ‘between those separated from the means of production’ (2013b: 46; see also Parry, 2019).

4 This was of course also part of the Nehruvian dream of absorbing the dispossessed into public sector industrial employment, which ‘would provide a beacon in terms of pay and conditions for the industrial working class as a whole’ (Parry, 2013a: 371). Instead, however, as this article too illustrates, public sector employees ‘became a privileged aristocracy of labour cocooned from the rest in a privileged enclave that is largely cut off from other fractions of labour’ (Parry, 2013a: 371).

5 Land acquisition for coal in India has been supported by legal provisions such as the 1894 Land Acquisition Act and, more specifically, the 1957 Coal Bearing Areas (Acquisition & Development) Act. The latter is also exempt from the amended 2013 Land Acquisition Act that seeks to provide fair compensation to dispossessed families.

6 For CIL projects, this was, in 1982, 3 acres, later reduced to 2 acres. Dharutar households that were dispossessed of less than 3 acres in 1982 were only entitled to a relatively small sum in monetary compensation.
